# *Magnolia* Extract (BL153) Ameliorates Kidney Damage in a High Fat Diet-Induced Obesity Mouse Model

**DOI:** 10.1155/2013/367040

**Published:** 2013-11-28

**Authors:** Wenpeng Cui, Yangwei Wang, Qiang Chen, Weixia Sun, Lu Cai, Yi Tan, Ki-Soo Kim, Ki Ho Kim, Young Heui Kim

**Affiliations:** ^1^The Second Hospital of Jilin University, Changchun 130041, China; ^2^The Kosair Children's Hospital Research Institute, Department of Pediatrics of the University of Louisville, Louisville, KY 20202, USA; ^3^School of Public Health, Jilin University, Changchun 130021, China; ^4^The First Hospital of Jilin University, Changchun 130021, China; ^5^Bioland Biotec Co., Ltd., Zhangjiang Modern Medical Device Park, Pudong, Shanghai 201201, China; ^6^Bioland R&D Center, 59 Songjeongni 2-gil, Byeongcheon, Dongnam, Cheonan, Chungnam 330-863, Republic of Korea

## Abstract

Accumulating evidence demonstrated that obesity is a risk factor for renal structural and functional changes, leading to the end-stage renal disease which imposes a heavy economic burden on the community. However, no effective therapeutic method for obesity-associated kidney disease is available. In the present study, we explored the therapeutic potential of a *magnolia* extract (BL153) for treating obesity-associated kidney damage in a high fat diet- (HFD-) induced mouse model. The results showed that inflammation markers (tumor necrosis factor-**α** and plasminogen activator inhibitor-1) and oxidative stress markers (3-nitrotyrosine and 4-hydroxy-2-nonenal) were all significantly increased in the kidney of HFD-fed mice compared to mice fed with a low fat diet (LFD). Additionally, proteinuria and renal structure changes in HFD-fed mice were much more severe than that in LFD-fed mice. However, all these alterations were attenuated by BL153 treatment, accompanied by upregulation of peroxisome proliferator-activated receptor-**γ** coactivator-1**α** (PGC-1**α**) and hexokinase II (HK II) expression in the kidney. The present study indicates that BL153 administration may be a novel approach for renoprotection in obese individuals by antiinflammation and anti-oxidative stress most likely via upregulation of PGC-1**α** and HK II signal in the kidney.

## 1. Introduction

The International Obesity Task Force has determined that the global numbers of adults and school-aged children considered obese in 2010 were 600 million and 50 million, respectively. As the worldwide prevalence of obesity is increasing, more attention has been given to obesity-related complications, including kidney disease. The causative relationship between obesity and proteinuria was first reported in 1974 [1]. After that, accumulating evidence supported that obesity is an independent risk factor for renal structural and functional changes, leading to end-stage renal disease which brings the society a heavy economic burden [2, 3]. However, no effective medicine treating this disease is available.

Insulin resistance and compensatory hyperinsulinemia are hallmarks of obesity. Several distinct pathways through which insulin resistance and hyperinsulinemia lead to renal damage have been reported by overwhelming background studies, such as insulin-like growth factor 1 upregulation [4] and renin-angiotensin system activation [5]. However, the mechanism underlying the generation of insulin resistance in obesity has not been fully understood. Recently, inflammation and oxidative stress have emerged to be involved in provoking insulin resistance in obesity-associated kidney damage [6, 7]. Therefore, alleviating inflammation and oxidative stress seems to be a potential therapeutic method for this disease.

Phytotherapy has been used for human disease as an alternative or complement to allopathic medicines for several centuries [8, 9]. One of the representative medicinal plants is the *Magnolia* genus, which is mainly distributed in East and Southeast Asia. Up till now, more than 250 kinds of ingredients have been isolated from the cones, bark, and leaves of the *Magnolia* genus, such as magnolol, honokiol, 4-O-methylhonokiol, and obovatol [10]. The medicinal use of this species is attributed to its different pharmacological effects, including anti-inflammation [11, 12] and antioxidative stress [13, 14]. In an early study, Munroe et al. found that honokiol administration obviously reduced airway hyperresponsiveness in ovalbumin-induced allergic asthma mice, accompanied by proinflammatory cytokines significantly reduction [15]. Subsequent observation suggested that *Magnolia grandiflora* L. flower extract exerted antioxidant capacities by depleting cellular reactive oxygen species in a dose-dependent pattern [16]. More recently, using a high fat diet- (HFD-) fed mouse model, Kim et al. found that long-term supplementation of honokiol and magnolol attenuated body fat accumulation, insulin resistance, and adipose inflammation [17]. However, it was unclear whether a renal protective effect would be seen in *magnolia* extract-treated obese mice.

Therefore, the current study was undertaken to study the effects of *magnolia* extract (BL153) on renal injury in an HFD-induced obesity mouse model. In addition, to further explore the possible mechanism, some candidate molecules involved in the renal inflammation, oxidative stress, and metabolism regulation were also detected.

## 2. Results

### 2.1. BL153 Decreases Obesity-Induced Renal Dysfunction and Structure Changes

**Figure 1:**
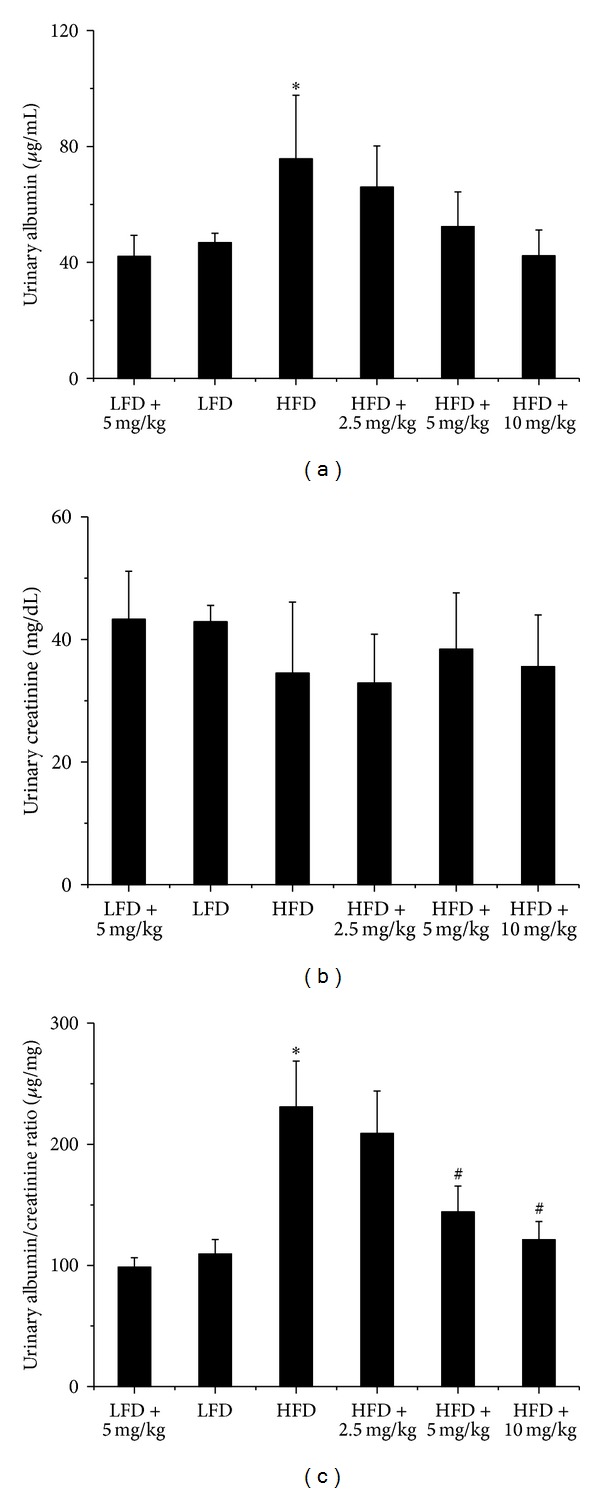
BL153 prevented obesity-induced renal functional changes. Male C57/BL6/J mice at 8 weeks of age were fed either a LFD (10% kcal as fat) or a HFD (60% kcal as fat) with or without indicated dose of BL153 (2.5 mg/kg, 5 mg/kg or 10 mg/kg) for 6 months. Urine samples were collected and then all mice were sacrificed for study. Urinary albumin and creatinine levels were examined to reflect renal function. *n* = 5; *P < 0.05 versus LFD group; ^#^ *P* < 0.05 versus HFD group.

To evaluate renal function, urinary albumin and urinary creatinine were detected, and based on which, urinary albumin-to-creatinine ratio (ACR) was calculated. Although remarkably elevated in HFD-fed mice at the end of experiment, ACR was significantly reduced after administration with BL153 at the dose of 5 mg/kg or 10 mg/kg for six months (Figure 1).

**Figure 2:**
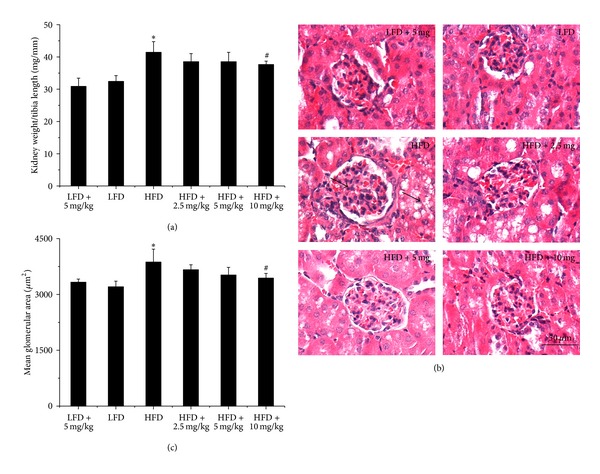
BL153 prevented obesity-induced renal structural changes. Kidney weight was normalized by tibia length (a). Renal pathology was examined by using hematoxylin and eosin staining (b), and the arrows indicated glomerular enlargement and renal tubular epithelium damage. Qualitative analysis for glomerular enlargement was indicated by mean glomerular area (c). *n* = 5; *P < 0.05 versus LFD group; ^#^ *P* < 0.05 versus HFD group.

Kidney weight to tibia length ratio (Figure 2(a)) and kidney hematoxylin and eosin (H&E) staining were undertaken to evaluate the protective effects of BL153 on obesity-induced kidney structure changes. As shown in Figure 2, kidney weight to tibia length ratio was increased in HFD-fed mice compared with low fat diet- (LFD-) fed mice. Additionally, kidney histopathological alterations in HFD-fed mice were mild at the end of experiment, such as glomerular enlargement and renal tubular epithelium damage. However, all these structure alterations were alleviated after six months of BL153 treatment at a dose of 5 mg/kg or 10 mg/kg (Figure 2).

### 2.2. BL153 Attenuates Obesity-Induced Renal Inflammation and Oxidative Stress

**Figure 3:**
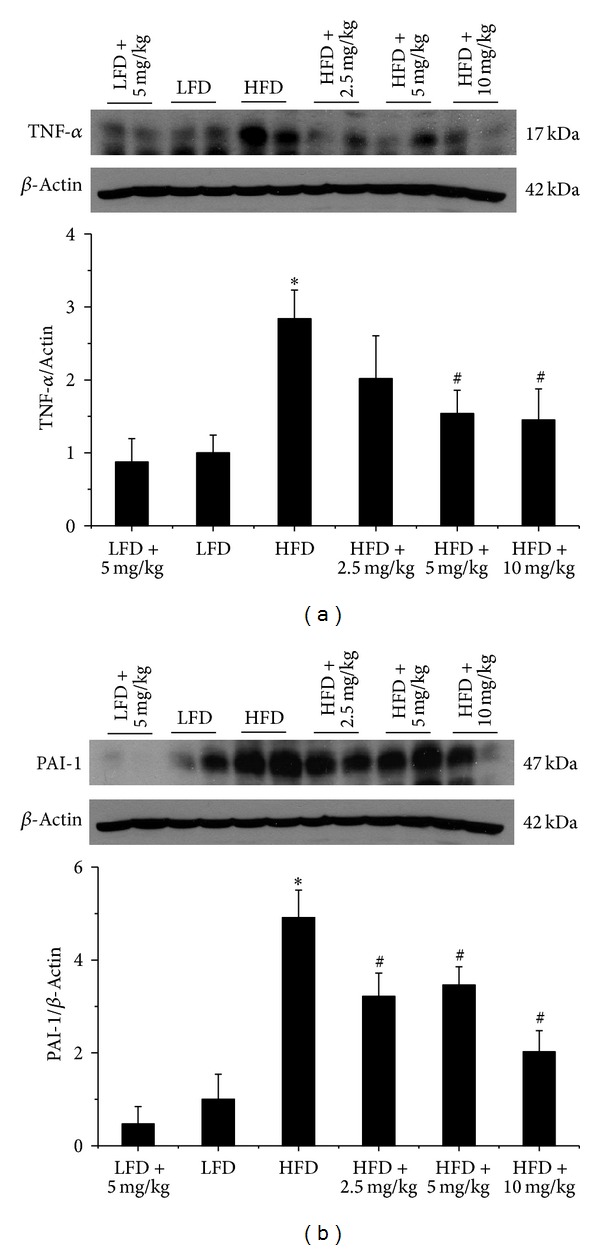
BL153 ameliorated obesity-induced renal inflammation. Western blot assay were performed for measuring the expression of inflammatory cytokines and TNF-*α* (a) and PAI-1 (b). *n* = 5; *P < 0.05 versus LFD group; ^#^ *P* < 0.05 versus HFD group.

Inflammation and oxidative stress play an important role in obesity-induced renal damage; hence, we performed western blot assay for the renal expression of inflammatory cytokines, tumor necrosis factor-alpha (TNF-*α*, Figure 3(a)), and plasminogen activator inhibitor-1 (PAI-1, Figure 3(b)), which were also well accepted as adipocytokines or adipokines [18]. Both of them were significantly increased in HFD mice compared with LFD mice; however, this trend was partially abolished by BL153 treatment (Figure 3).

**Figure 4:**
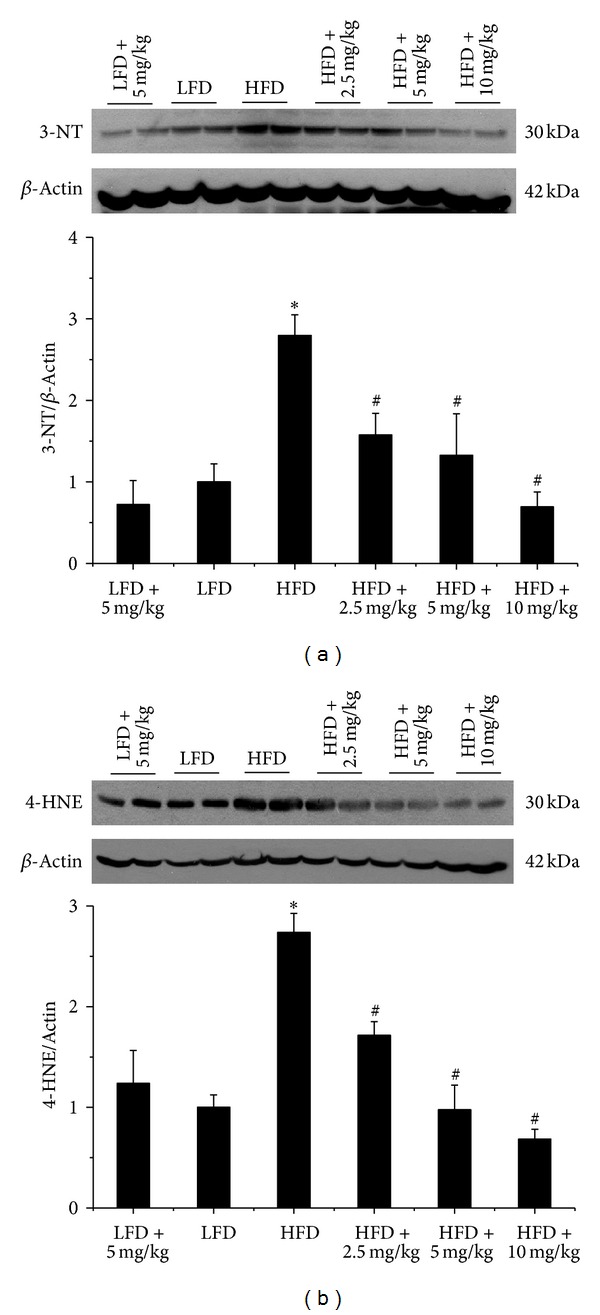
BL153 ameliorated obesity-induced renal oxidative stress. Western blot assay were performed for measuring the oxidative damage including the accumulation of 3-NT (a) and 4-HNE (b). *n* = 5; *P < 0.05 versus LFD group; ^#^ *P* < 0.05 versus HFD group.

In addition, we also investigated the protective effects of BL153 on obesity-induced oxidative stress by examining 3-nitrotyrosine (3-NT, Figure 4(a)) as an index of nitrosative damage and 4-hydroxy-2-nonenal (4-HNE, Figure 4(b)) as an index of oxidative damage in the next study. Similarly, increased kidney 3-NT and 4-HNE expression caused by HFD feeding were all prevented by BL153 treatment (Figure 4).

### 2.3. Protective Effects of BL153 on Obesity-Induced Renal Damage Is Associated with the Upregulation of Renal Peroxisome Proliferator-Activated Receptor-*γ* Coactivator-1*α* (PGC-1*α*), NAD(P)H Quinone Oxidoreductase 1 (NQO1), and Hexokinase II (HK II) Expression

**Figure 5:**
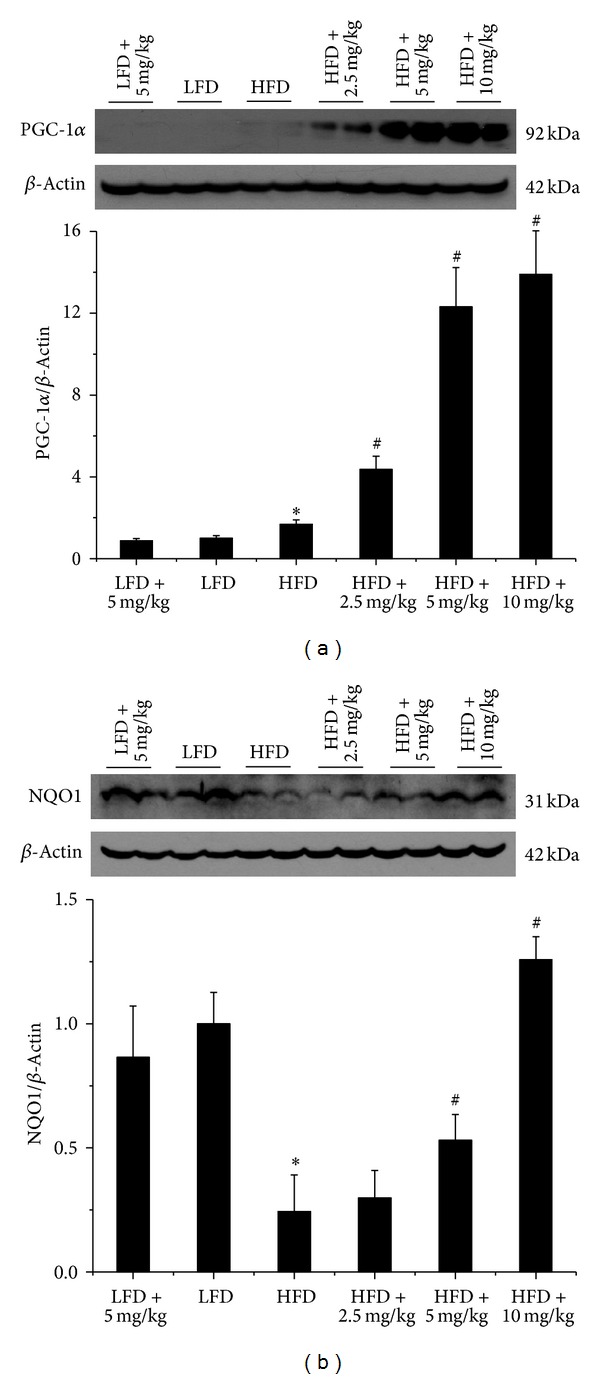
BL153 upregulated renal PGC-1*α* and NQO1 expression. PGC-1*α* and NQO1 expression was detected by western blot assay. *n* = 5; *P < 0.05 versus LFD group; ^#^ *P* < 0.05 versus HFD group.

The next study was to explore the possible mechanism by which BL153 attenuates kidney inflammation and oxidative stress in obesity. As shown in Figure 5, kidney PGC-1*α* was slightly increased in HFD mice compared with LFD mice, and it was further increased after six-month BL153 treatment at indicated doses (Figure 5(a)). Additionally, kidney NQO1 was significantly decreased in HFD mice compared with LFD mice; however, it was increased by BL153 administration (Figure 5(b)).

**Figure 6:**
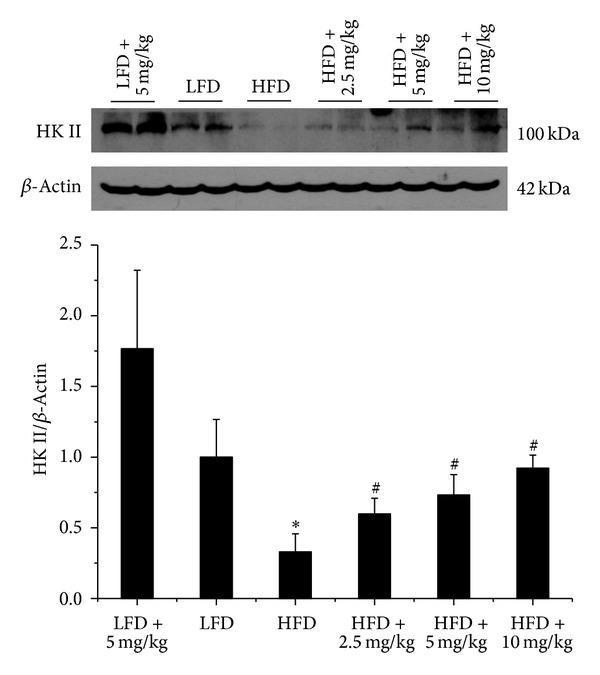
BL153 upregulated renal HK II expression. HK II expression was detected by western blot assay. *n* = 5; *P < 0.05 versus LFD group; ^#^ *P* < 0.05 versus HFD group.

Another candidate molecule is HK II. As shown in Figure 6, HK II protein expression was decreased in kidney of HFD group mice compared with LFD group mice; however, it was almost back to normal level by BL153 administration at indicated doses (Figure 6).

## 3. Discussion

In current study, we provide the first evidence that *magnolia* extract, BL153, attenuates obesity-associated renal structural and functional changes in an HFD-induced obesity mouse model. Importantly, BL153 treatment attenuated obesity caused renal inflammation and oxidative stress, which was likely due to the ability of BL153 to increase PGC-1*α* and HK II expression in kidney.

Obesity is considered as a state of chronic low-grade systemic inflammation and oxidative stress, which interact with each other and cause a vicious circle, promoting the development of insulin resistance [19, 20]. This concept has been proven by numerous studies. For instance, using human visceral adipose cells, Vazquez-Carballo et al. found that TNF-*α* treatment leads to insulin resistance upon glucose uptake, glucose transporter 4 translocation, and insulin signaling [21]. Experimental data and human studies indicated that 4-HNE, a major oxidation product of membrane lipids, impaired insulin signaling and disrupted the insulin biological activity in skeletal muscle [22]. Consistent with these reports, our present study found that inflammatory markers, like TNF-*α* and PAI-1, and oxidative stress markers, like 3-NT and 4-HNE, were elevated in HFD-fed mice, accompanied by insulin resistance as we reported recently (Sun et al., unpublished paper).

Our data demonstrated for the first time that these alterations could be reversed by BL153 administration. Several previous studies about *magnolia* extract obtained similar conclusions. Purified from the *Magnolia officinalis*, magnolol was found to exert an anti-inflammatory property via repressing lipopolysaccharide-induced Toll-like receptor 4 expression, subsequent nuclear factor kappa-B (NF-*κ*B), and MAPK signaling pathway in uterine epithelial cells [23]. Furthermore, *in vivo* study indicated that honokiol, another compound isolated from the Magnolia herb, modulated inflammation-associated cytokines, such as interleukin-1*β*, interleukin-6, TNF-*α*, and monocyte chemoattractant protein-1, through activation of NF-*κ*B [24]. Besides anti-inflammation properties, *Magnolia* extract has also been found to take part in ameliorating oxidative stress through different pathways. For example, 4-O-methylhonokiol, a novel compound isolated from *Magnolia officinalis*, prevents the development and progression of Alzheimer's disease by improving oxidative stress through a p38 MAPK-dependent pathway [25]. The beneficial effects of magnolol on learning and memory abilities were reported to be associated with superoxide dismutase (SOD) restoration in a scopolamine-induced mouse model [26]. However, how does *magnolia* extract modulate kidney inflammation and oxidative stress in obesity status is still unclear.

**Figure 7:**
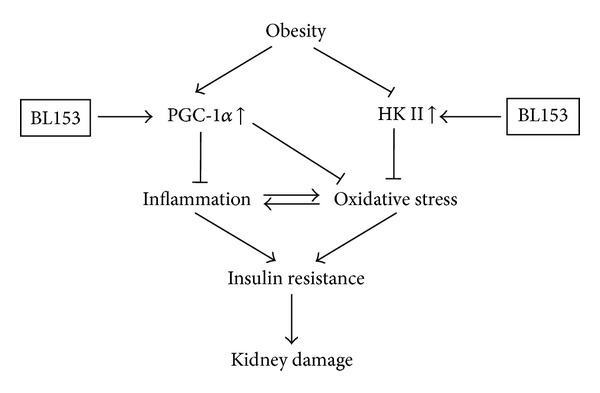
A proposed mechanism by which BL153 attenuates kidney damage during obesity is illustrated.

In our present study, a novel finding was that PGC-1*α* was increased by more than 10-folds after BL153 treatment. As a well-known insulin resistance regulator, PGC-1*α* also has been reported to exert anti-inflammation effects by multiple mechanisms [27–29]. For instance, overexpression of PGC-1*α* in cultured vascular smooth muscle cells leads to decreased reactive oxygen species generation by the increased expression of SOD2 in the mitochondria. The knockdown of PGC-1*α* by specific small interfering RNA greatly reduced mitochondrial antioxidative protein expression [29]. In this study, we demonstrated that NQO1, but not SOD2 (data not shown), was significantly increased by BL153 administration (Figure 5(b)). Taken together, it is possible that *magnolia* extract (BL153) attenuates kidney inflammation and oxidative stress via increasing PGC-1*α*-mediated various antioxidative protein expressions under different conditions. Moreover, our results showed that PGC-1*α* was slightly elevated in the kidney of HFD-fed mice. Similar elevation was also found in the pancreas islets of type 2 diabetic rats [30], while other observation declared a reduction of PGC-1*α* in the adipose tissue of insulin-resistant and obesity individuals [31]. We assumed that the increasing PGC-1*α* found in HFD-fed mouse kidney was due to a positive feedback from insulin resistance to overcome the impairment of local homeostasis, as illustrated in Figure 7.

Another important observation in this study was that kidney HK II expression was lower in HFD group mice than that in LFD group mice. This finding was supported by a previous human study in obesity and type 2 diabetes individuals [32]. However, after treatment with BL153, HK II expression was upregulated in HFD-fed mouse kidney, accompanied by renal oxidative stress alleviation. Once worked as a pivotal mediator in glycolysis by catalyzing the phosphorylation of glucose to generate glucose 6-phosphate [32], HK II has emerged to play a role in oxidative stress recently [33, 34]. Based on these, we hypothesized that increasing HK II expression might be another mechanism underlying renoprotection by BL153 in obesity-induced kidney injury. On the other hand, overexpression of HK II is involved in cancer cell proliferation and migration [35]. Our data showed that BL153 administration did not affect HK II expression level in LFD group mice; moreover, BL153 administration in HFD group mice only increased HK II expression back to the normal level, even at a high dose (10 mg/kg), indicating the safety of BL153, as illustrated in Figure 7.

There is one limitation in our study. To make sure the crucial role of PGC-1*α* and HK II in BL153-associated kidney prevention during obesity, PGC-1*α* and HK II knockout animals should be used. We hope that further research will confirm our hypothesis.

The findings in the present study were relevant but distinct from our previous studies [36, 37], in which both inflammation and oxidative stress have been found to be significantly increased in kidney in type 1 diabetic mouse. In addition, ameliorating inflammation and oxidative stress by using MG132 or sulforaphane showed beneficial effects in diabetic nephropathy [36, 37]. MG132 is a peptide aldehyde proteasome inhibitor and exerts its therapeutic effects by reducing the degradation of ubiquitin-conjugated proteins such as nuclear-factor-E2-related-factor-2 (Nrf2), an important transcription factor regulating cellular defenses against reactive oxygen species, through inhibiting activity of the *β* subunits of the core particle of 26S proteasome [38]. Sulforaphane, isolated from cabbage and broccoli, is another Nrf2 activator and regulates Nrf2 by disassociating Nrf2 from its inhibitor Kelch-like ECH-associated protein 1 and subsequently facilitating Nrf2 into the nucleus [39]. However, Zhang et al. found that increasing Nrf2 activity did not prevent diet-induced obesity and had limited effects on lipid metabolism [40]. Moreover, the concept of more-harm-than-benefit effect of Nrf2 on obesity has emerged. Xu et al. demonstrated that enhanced Nrf2 activity even impaired insulin signaling, prolonged hyperglycemia in response to glucose challenge, and induced insulin resistance in leptin-deficient obesity [41]. Therefore, compounds such as BL153, with therapeutic efficacy and distinct mechanisms from MG132 and sulforaphane, have great potential for treatment of obesity-associated nephropathy.

In summary, our results first demonstrate a therapeutic potential of BL153 in treating obesity-induced kidney disease. BL153 administration in an obese state attenuates kidney inflammation and oxidative stress, leading to improved renal pathological and functional alterations. Furthermore, our data suggested that the possible mechanism through which BL153 ameliorates renal inflammation and oxidative stress is PGC-1*α* and HK II upregulation in kidney. Taken together, the present study declares that BL153 administration may be a novel approach for renoprotection in obesity individuals.

## 4. Material and Methods

### 4.1. *Magnolia* Extract (BL153)

The bark of *Magnolia officinalis* were purchased from Kyungdong market, Seoul, Korea and was taxonomically identified by Dr. Ban Yeon Hwang at the Research Institute of Drug Resource, Chungbuk National University (Cheongju, Korea). A voucher specimen was deposited at the Herbarium of Chungbuk National University, Chungbuk, Korea. The bark of *M. officinalis* was dried in the shade at room temperature and stored in a dark, cold room until use. The air-dried bark of *M. officinalis* (3 kg) was cut into pieces and extracted twice with 95% (v/v) ethanol (4 times as much as the weight of the dried plants) for 3 days at room temperature. After filtration through the 400-mesh filter cloth, the filtrate was filtered again through filter paper (Whatman, no. 5) and concentrated under reduced pressure to obtain viscous dark-brown residue (360 g, BL153). The ethanol extract of *M. officinalis* (BL153) was analyzed by HPLC to ensure mainly that it is containing 14.8% of 4-O-methylhonokiol, 14.2% of honokiol, and 12.0% of magnolol, which were in agreement with previously published data [42].

### 4.2. Experimental Animals and Protocols

All experiments involving mice conformed to the National Institutes of Health Guide for the Care and Use of Laboratory Animals and were approved by the University of Louisville Institutional Animal Care and Use Committee.

Male C57/BL6/J mice at 8 weeks of age were purchased from the Jackson Laboratory and housed in the University of Louisville Research Resources Center at 22°C with a 12-hour light/dark cycle. Thirty mice were randomly divided into six groups (*n* = 5) and fed either an LFD (10% kcal as fat; D12450B, Research Diets Inc. NJ) or an HFD (60% kcal as fat; D12492B, Research Diets Inc. NJ) with or without BL153 for six months. (1) LFD + 5 mg/kg group: mice were fed an LFD and supplemented with BL153 at the dose of 5 mg/kg; (2) LFD group: mice were fed an LFD and supplemented with 0.5% ethanol; (3) HFD group: mice were fed an HFD and supplemented with 0.5% ethanol; (4) HFD + 2.5 mg/kg group: mice were fed an HFD and supplemented with BL153 at the dose of 2.5 mg/kg; (5) HFD + 5 mg/kg group: mice were fed an HFD and supplemented with BL153 at the dose of 5 mg/kg; and (6) HFD + 10 mg/kg group: mice were fed an HFD and supplemented with BL153 at the dose of 10 mg/kg. Selection of 5 mg/kg and 10 mg/kg for the present study was based on a previous study [42], where treatment with BL153 at these two dose levels for a week showed a significantly protective effect. Since here the treatment is longer than that, we also included one low dose of BL153 at 2.5 mg/kg.

For preparing BL153 gavage solution, different doses of BL153 were dissolved into 100% ethanol first and then diluted with ddH_2_O into final concentration of BL153 at 1.0 mg/mL (high dose group), 0.5 mg/mL (middle dose group), and 0.25 mg/mL (low dose group) with final concentration of ethanol at 0.05%, respectively. Therefore, the gavage volume was 1% (mL/g) of mouse body weight (e.g., 25 g mouse should be given 250 *μ*L). Control groups were given same volume of ddH_2_O with 0.05% ethanol. During the six-month feeding, body weight was measured every month, and the gavage volume was justified based on the body weight change. At the end of experiment, after urine samples were collected, all mice were sacrificed for further analysis.

### 4.3. Urinary Albumin Assay

Urine samples were collected at the end of experiment. Urine albumin (Bethyl Laboratories, Montgomery, TX) and urinary creatinine (BioAssay Systems, Hayward, CA) were measured according to the manufacturers' instructions. ACR was calculated as ACR = urine albumin/urine creatinine (*μ*g/mg), as we previously described [36].

### 4.4. Kidney Histopathological Examination

Kidney tissue was fixed overnight in 10% phosphate-buffered formalin and then dehydrated in a graded alcohol series, cleared with xylene, embedded in paraffin, and sectioned at 5 *μ*m thickness for pathological staining. To examine overall morphology, paraffin sections were dewaxed for H&E staining. Mean glomerular area was also measured to evaluate the glomerular enlargement.

### 4.5. Western Blot Assay

Kidney tissues were homogenized in RIPA buffer and total protein was extracted. Western blot assay was performed as previously reported [37]. Briefly, protein was separated on 10% SDS-PAGE gels and transferred to nitrocellulose membranes (Bio-Rad, Hercules, CA). The latter were blocked with 5% milk, followed by incubation with the following antibodies: TNF-*α*, PGC-1*α* (Abcam, Cambridge, MA), PAI-1 (BD Bioscience, San Jose, CA), 3-NT (Millipore, Billerica, MA), 4-HNE (Alpha Diagnostic International, San Antonio, TX), HK II, *β*-actin, and NQO1 (SantaCruz Biotechnology, Santa Cruz, CA). After those membranes were washed with Tris-buffered saline (pH 7.2) containing 0.05% Tween 20 and incubated with the appropriate secondary antibodies. Protein bands were visualized using enhanced chemiluminescence (Thermo scientific, Rockford, IL).

### 4.6. Statistical Analysis

Data were collected from five animals for each group and presented as means ± SD. We used Image Quant 5.2 to analyze western blotting. Comparisons between groups were performed by one-way ANOVA, followed by Tukey's post hoc test. Statistical analysis was performed with Origin 7.5 Laboratory data analysis and graphing software. Statistical significance was considered as *P* < 0.05.

## References

[1] Weisinger JR, Kempson RL, Eldridge FL, Swenson RS (1974). The nephrotic syndrome: a complication of massive obesity. *Annals of Internal Medicine*.

[2] Spoto B, Mattace-Raso F, Sijbrands E (2012). The fat-mass and obesity-associated gene (Fto) predicts mortality in chronic kidney disease of various severity. *Nephrology Dialysis Transplantation*.

[3] Cui W, Maimaitiyiming H, Qi X, Norman H, Wang S (2013). Thrombospondin 1 mediates renal dysfunction in a mouse model of high-fat diet-induced obesity. *American Journal of Physiology*.

[4] Sesti G, Succurro E, Arturi F (2011). IGF-1 levels link estimated glomerular filtration rate to insulin resistance in obesity: a study in obese, but metabolically healthy, subjects and obese, insulin-resistant subjects. *Nutrition, Metabolism and Cardiovascular Diseases*.

[5] Weisinger RS, Stanley TK, Begg DP, Weisinger HS, Spark KJ, Jois M (2009). Angiotensin converting enzyme inhibition lowers body weight and improves glucose tolerance in C57BL/6J mice maintained on a high fat diet. *Physiology and Behavior*.

[6] Asghar M, Monjok E, Kouamou G, Ohia SE, Bagchi D, Lokhandwala MF (2007). Super CitriMax (HCA-SX) attenuates increases in oxidative stress, inflammation, insulin resistance, and body weight in developing obese Zucker rats. *Molecular and Cellular Biochemistry*.

[7] Li Y, Tong X, Rumala C, Clemons K, Wang S (2011). Thrombospondin1 deficiency reduces obesity-associated inflammation and improves insulin sensitivity in a diet-induced obese mouse model. *PLoS ONE*.

[8] Singh H (2012). Traditional phytotherapy for the treatment of hydrocele in Odisha, India. *Ancient Science of Life*.

[9] Cavero RY, Akerreta S, Calvo MI (2013). Medicinal plants used for dermatological affections in navarra and their pharmacological validation. *Journal of Ethnopharmacology*.

[10] Lee Y-J, Lee YM, Lee C-K, Jung JK, Han SB, Hong JT (2011). Therapeutic applications of compounds in the Magnolia family. *Pharmacology and Therapeutics*.

[11] Walker JM, Maitra A, Walker J (2013). Identification of *Magnolia officinalis* L. Bark extract as the most potent anti-inflammatory of four plant extracts. *American Journal of Chinese Medicine*.

[12] Kuo WL, Chung CY, Hwang TL, Chen JJ (2013). Biphenyl-type neolignans from *Magnolia officinalis* and their anti-inflammatory activities. *Phytochemistry*.

[13] Shih HC, Hwang TL, Chen HC (2013). Honokiol dimers and magnolol derivatives with new carbon skeletons from the roots of *Magnolia officinalis* and their inhibitory effects on superoxide anion generation and elastase release. *PLoS ONE*.

[14] Yu S-X, Yan R-Y, Liang R-X, Wang W, Yang B (2012). Bioactive polar compounds from stem bark of *Magnolia officinalis*. *Fitoterapia*.

[15] Munroe ME, Businga TR, Kline JN, Bishop GA (2010). Anti-inflammatory effects of the neurotransmitter agonist Honokiol in a mouse model of allergic asthma. *Journal of Immunology*.

[16] Huang HC, Hsieh WY, Niu YL, Chang TM (2012). Inhibition of melanogenesis and antioxidant properties of magnolia grandiflora L. Flower extract. *BMC Complementary and Alternative Medicine*.

[17] Kim YJ, Choi MS, Cha BY (2013). Long-term supplementation of honokiol and magnolol ameliorates body fat accumulation, insulin resistance, and adipose inflammation in high-fat fed mice. *Molecular Nutrition & Food Research*.

[18] Shimomura I, Funahashi T, Takahashi M (1996). Enhanced expression of PAI-1 in visceral fat: possible contributor to vascular disease in obesity. *Nature Medicine*.

[19] Xu H, Barnes GT, Yang Q (2003). Chronic inflammation in fat plays a crucial role in the development of obesity-related insulin resistance. *Journal of Clinical Investigation*.

[20] Furukawa S, Fujita T, Shimabukuro M (2004). Increased oxidative stress in obesity and its impact on metabolic syndrome. *Journal of Clinical Investigation*.

[21] Vazquez-Carballo A, Ceperuelo-Mallafre V, Chacon MR (2013). Tweak prevents Tnf-Alpha-induced insulin resistance through Pp2a activation in human adipocytes. *American Journal of Physiology*.

[22] Pillon NJ, Croze ML, Vella RE, Soulère L, Lagarde M, Soulage CO (2012). The lipid peroxidation by-product 4-hydroxy-2-nonenal (4-HNE) induces insulin resistance in skeletal muscle through both carbonyl and oxidative stress. *Endocrinology*.

[23] Luo J, Xu Y, Zhang M, Gao L, Fang C, Zhou C (2013). Magnolol inhibits Lps-induced inflammatory response in uterine epithelial cells: magnolol inhibits Lps-induced inflammatory response. *Inflammation*.

[24] Chiang J, Shen Y-C, Wang Y-H (2009). Honokiol protects rats against eccentric exercise-induced skeletal muscle damage by inhibiting NF-*κ*B induced oxidative stress and inflammation. *European Journal of Pharmacology*.

[25] Lee YK, Choi IS, Ban JO (2011). 4-O-methylhonokiol attenuated *β*-amyloid-induced memory impairment through reduction of oxidative damages via inactivation of p38 MAP kinase. *Journal of Nutritional Biochemistry*.

[26] Li YS, Hong YF, He J (2013). Effects of magnolol on impairment of learning and memory abilities induced by scopolamine in mice. *Biological and Pharmaceutical Bulletin*.

[27] Koo S-H, Satoh H, Herzig S (2004). PGC-1 promotes insulin resistance in liver through PPAR-*α*-dependent induction of TRB-3. *Nature Medicine*.

[28] Hara K, Tobe K, Okada T (2002). A genetic variation in the PGC-1 gene could confer insulin resistance and susceptibility to Type II diabetes. *Diabetologia*.

[29] Qu A, Jiang C, Xu M (2009). PGC-1*α* attenuates neointimal formation via inhibition of vascular smooth muscle cell migration in the injured rat carotid artery. *American Journal of Physiology*.

[30] Yoon JC, Xu G, Deeney JT (2003). Suppression of *β* cell energy metabolism and insulin release by PGC-1*α*. *Developmental Cell*.

[31] Semple RK, Crowley VC, Sewter CP (2004). Expression of the thermogenic nuclear hormone receptor coactivator PGC-1*α* is reduced in the adipose tissue of morbidly obese subjects. *International Journal of Obesity*.

[32] Pendergrass M, Koval J, Vogt C (1998). Insulin-induced hexokinase II expression is reduced in obesity and NIDDM. *Diabetes*.

[33] Wu R, Wyatt E, Chawla K (2012). Hexokinase Ii knockdown results in exaggerated cardiac hypertrophy via increased ros production. *EMBO Molecular Medicine*.

[34] Mailloux RJ, Dumouchel T, Aguer C, Dekemp R, Beanlands R, Harper M-E (2011). Hexokinase II acts through UCP3 to suppress mitochondrial reactive oxygen species production and maintain aerobic respiration. *Biochemical Journal*.

[35] Jiang S, Zhang L-F, Zhang H-W (2012). A novel miR-155/miR-143 cascade controls glycolysis by regulating hexokinase 2 in breast cancer cells. *The EMBO Journal*.

[36] Cui W, Bai Y, Miao X (2012). Prevention of diabetic nephropathy by sulforaphane: possible role of Nrf2 upregulation and activation. *Oxidative Medicine and Cellular Longevity*.

[37] Cui W, Li B, Bai Y (2013). Potential role for Nrf2 activation in the therapeutic effect of Mg132 on diabetic nephropathy in Ove26 diabetic mice. *American Journal of Physiology*.

[38] Cui W, Bai Y, Luo P, Miao L, Cai L (2013). Preventive and therapeutic effects of Mg132 by activating Nrf2-are signaling pathway on oxidative stress-induced cardiovascular and renal injury. *Oxidative Medicine and Cellular Longevity*.

[39] Guerrero-Beltrán CE, Calderón-Oliver M, Pedraza-Chaverri J, Chirino YI (2012). Protective effect of sulforaphane against oxidative stress: recent advances. *Experimental and Toxicologic Pathology*.

[40] Zhang YK, Wu KC, Liu J, Klaassen CD (2012). Nrf2 deficiency improves glucose tolerance in mice fed a high-fat diet. *Toxicology and Applied Pharmacology*.

[41] Xu J, Kulkarni SR, Donepudi AC, More VR, Slitt AL (2012). Enhanced Nrf2 activity worsens insulin resistance, impairs lipid accumulation in adipose tissue, and increases hepatic steatosis in leptin-deficient mice. *Diabetes*.

[42] Lee YK, Yuk DY, Kim TI (2009). Protective effect of the ethanol extract of *Magnolia officinalis* and 4-O-methylhonokiol on scopolamine-induced memory impairment and the inhibition of acetylcholinesterase activity. *Journal of Natural Medicines*.

